# Effect of Subconjunctival Injection of Canine Adipose-Derived Mesenchymal Stem Cells on Canine Spontaneous Corneal Epithelial Defects

**DOI:** 10.3390/ani14223270

**Published:** 2024-11-13

**Authors:** Pechchalee Kengkla, Yaowalak Panyasing, Aree Thayananuphat, Nalinee Tuntivanich

**Affiliations:** 1 Department of Veterinary Surgery, Faculty of Veterinary Science, Chulalongkorn University, Bangkok 10330, Thailand; geegee3413@gmail.com; 2 Department of Pathology, Faculty of Veterinary Science, Chulalongkorn University, Bangkok 10330, Thailand; yaowalak.p@chula.ac.th; 3 Department of Companion Animal Clinical Sciences, Faculty of Veterinary Medicine, Kasetsart University, Bangkok 10900, Thailand; areethaya@gmail.com

**Keywords:** canine adipose-derived mesenchymal stem cell, dog, subconjunctival injection, spontaneous chronic corneal epithelial defects, tumor necrosis factor-alpha, vascular endothelium growth factor-A

## Abstract

Spontaneous chronic corneal epithelial defects (SCCEDs) are characterized by nonadherent corneal epithelium leading to poor attachment to the corneal stroma. The objective of this study was to characterize corneal outcomes concurrently with the quantification of tumor necrosis factor-alpha (TNF-α) and vascular endothelial growth factor-A (VEGF-A) in tear fluid after the subconjunctival injection of canine adipose-derived mesenchymal stem cells (cAD-MSCs) in canine SCCEDs. Ten eyes with SCCEDs, which were nonresponsive to two rounds of diamond burr debridement, were included in this study. All eyes received a single subconjunctival injection of 1 × 10^6^ cAD-MSCs. Ophthalmic examinations were performed before treatment and on day 7, 14, and 21 after treatment. Tear samples were collected for the quantification of TNF-α and VEGF-A concentrations by a canine multiplex immunoassay. Nine out of ten eyes revealed complete healing by day 21. The mean healing time was 10.89 ± 1.7 days. All eyes showed a decreased degree of ocular discomfort, in accordance with the degree of corneal characteristics. The concentrations of VEGF-A significantly reduced from pre-treatment (4334.91 ± 1275.92 pg/mL) to day 21 post-treatment (3064.61 ± 1028.66 pg/mL). No significant difference in TNF-α concentration was observed before/after treatment. In conclusion, the single use of a subconjunctival injection of cAD-MSCs could be used as an alternative treatment for canine SCCEDs.

## Simple Summary

Spontaneous chronic corneal epithelial defects are characterized by corneal epithelial dysmaturation and a superficial stromal hyalinized acellular zone that leads to poor attachment between the epithelium and anterior stroma. The conventional treatment of spontaneous chronic corneal epithelial defects involves topical therapy combined with corneal debridement. In cases that fail to achieve corneal healing via corneal debridement, surgical interventions under general anesthesia are required. Mesenchymal stem cells have been used for cell therapy of various corneal diseases in companion animals. In this study, the single use of a subconjunctival injection of canine adipose-derived mesenchymal stem cells provided satisfactory corneal outcomes in canine spontaneous chronic corneal epithelial defects that were nonresponsive to diamond burr debridement.

## 1. Introduction

Stem cell therapies are widely used in both human and veterinary regenerative medicine. Among the different types of post-natal stem cells, mesenchymal stem cells (MSCs) are considered an alternative cell-based therapy for the treatment of ocular diseases in humans and animals [1,2]. Stem cells can not only proliferate and differentiate, but they also continue proliferating in an undifferentiated state, maintaining the stem cell pool of the tissues [2]. They also have immunomodulatory and anti-inflammatory mechanisms that include the suppression of pro-inflammatory cytokines, the production of anti-inflammatory cytokines, and the inhibition of neutrophil, macrophage, and lymphocyte activation. Studies in a rat model of ocular alkali burn showed that the subconjunctival administration of bone marrow mesenchymal stem cells (BM-MSCs) significantly reduced the expression of pro-inflammatory cytokines such as TNF-α, interleukin-6 (IL-6), interleukin-1 (IL-1), monocyte chemoattractant protein-1 (MCP-1), and macrophage inflammatory protein-1 α (MIP-1α), as well as the infiltration of macrophages (CD86+ and CD45-positive cells) in the cornea [3,4]. Additionally, a study in mice demonstrated that the subconjunctival injection of mouse-derived BM-MSCs resulted in a reduced inflammatory response on the ocular surface and inhibited the infiltration of CD45-positive cells [5]. Recently, several studies using MSCs for corneal epithelial regeneration in animal models have been published. These demonstrated that not only are MSCs safe, but they also can promote the corneal healing process and epithelial regeneration, recover corneal transparency, and ultimately restore vision [2].

SCCEDs are characterized by corneal epithelial dysmaturation and a superficial stromal hyalinized acellular zone that leads to poor attachment between the epithelium and anterior stroma. This zone is presumed to be a barrier to the reformation of corneal adhesion complexes and normal basement membranes [6,7,8,9,10]. If left untreated, the defect can persist in association with severe ocular pain and a reduced quality of life. Many therapeutic methods have been proposed for the treatment of canine SCCEDs. Several topical therapies aiming to promote corneal wound healing of SCCEDs have been reported, including topical epidermal growth factor, polysulfated glycosaminoglycans, apoprotinin, substance P, insulin-like growth factor-1, and tetracycline [7,8,10,11]. However, topical treatments alone are still insufficient to achieve corneal healing. Instead, the stimulation of corneal epithelial regrowth through surgical intervention has been suggested. Mechanical debridement is commonly used as a conventional technique for the treatment of SCCEDs. This technique stimulates corneal healing by removing the abnormal epithelium and basement membrane as well as debris from the exposed stroma. This promotes epithelial cell proliferation and adhesion. However, the resolution rate of single dry debridement alone was approximately 50% [7,10]. One study reported that diamond burr debridement significantly decreased the thickness of the hyalinized acellular zone in the anterior stroma; therefore, it increased the overall healing rate to 92% [6]. In cases that fail to achieve corneal healing by debridement, surgical interventions such as grid keratotomy or superficial keratectomy with third eyelid flap are required. Although surgical interventions increase the chance of corneal healing, general anesthesia is involved [7,10].

In veterinary ophthalmology, MSCs have been used for cell therapy of various corneal diseases in experimental models. In diabetic mice, after mechanical removal of the corneal and limbal epithelium, subconjunctivally injected BM-MSCs decreased the epithelial defects and improved corneal re-epithelization as confirmed by the expression of Ki67 in the wound areas [12]. Yao et al. [13] studied the effect of BM-MSC administration in a chemical burn model of rat corneas. They applied two subconjunctival injections, immediately after the injury and again 3 days later. After 7 days, neovascularization was decreased as confirmed by the reduction in VEGF expression and the fast corneal epithelium recovery. Furthermore, a rabbit model of corneal alkali burn demonstrated that corneal epithelial defects, opacity, and neovascularization were reduced after the subconjunctival injection of cultured human AD-MSCs. Histological analysis revealed a significant increase in the layers of corneal epithelial cells, along with elevated levels of proliferation markers such as Cx43 and beta-catenin [14]. The combination of subconjunctival and topical administration of cAD-MSCs in various types of canine corneal ulcers, including anterior stromal ulcer (N = 9), posterior stromal ulcer (N = 9), descemetocele (N = 9), melting cornea (N = 6), and SCCEDs (N = 2), in 26 dogs revealed 84.6% complete corneal wound healing within 14 days after treatment. Among all corneal diseases, complete healing occurred in both eyes with SCCEDs [15]. While MSCs are gaining popularity in veterinary practice, there is limited study of MSC application in canine SCCEDs; the present study aimed to assess corneal outcomes and concentrations of tear fluid cytokines in canine eyes with SCCEDs receiving subconjunctival injection of cAD-MSCs.

## 2. Materials and Methods

### 2.1. Animals

This study was approved by the Institutional Animal Care and Use Committee (IACUC), Faculty of Veterinary Science, Chulalongkorn University (Approval ID:2131024). All dogs were clinical cases presenting with SCCEDs. Ten eyes from nine dogs, presented to the Ophthalmology Unit, Small Animal Teaching Hospital, Faculty of Veterinary Science, Chulalongkorn University, Bangkok, Thailand, were included in this study. All procedures were performed after obtaining approval from the owner via an informed consent form.

The dogs included in this study met the following criteria: (1) presence of SCCEDs with anterior stromal involvement; (2) history of two rounds of diamond burr debridement with no evidence of complete corneal healing; (3) absence of an underlying cause of the persistent corneal defect (e.g., eyelid or eyelash abnormalities) and significant ocular comorbidity disease (e.g., uveitis or glaucoma); and (4) Schirmer tear test (STT) value not less than 5 mm wetting per minute.

### 2.2. Ophthalmic Examinations

All the participating dogs underwent complete ophthalmic examinations at all time points before (day 0) and after treatment on days 7, 14, and 21. Ophthalmic examinations included neuro-ophthalmic reflexes (menace response, dazzle reflex, pupillary light response, and blink reflex), STT, a fluorescein staining test, tonometry (TonoVet Plus; Icare, Finland Oy, Helsinki, Finland), and slit lamp biomicroscopy (SL-2 handheld slit lamp; Kowa, Tokyo, Japan).

### 2.3. cAD-MSC Preparation

#### 2.3.1. Tissue Collection, Cell Isolation, Culture, and Cryopreservation

Adipose tissues were derived from a healthy four-year-old mixed-breed dog with the owner’s consent. A total of 20 g of subcutaneous adipose tissue was taken from the lumbar area, preserved in Dulbecco’s Phosphate-Buffered Saline (DPBS; Gibco^TM^, Thermo Fisher Scientific, Inc., Waltham, MA, USA), and delivered to the laboratory within 2 h at 4–8 °C. The tissue was washed with DPBS, minced, and digested with collagenase type I (Gibco^TM^, Thermo Fisher Scientific, Inc., Waltham, MA, USA) at 37 °C for 30 min. After neutralizing collagenase with culture medium, the tissue was filtered and centrifuged to isolate the stromal vascular fraction, which was washed with DPBS several times and plated at a density of 10^5^ cells/ T-25 flasks with Dulbecco’s Modified Eagle Medium (Gibco^TM^; Thermo Fisher Scientific, Inc., Waltham, MA, USA), 10% fetal calf serum (Gibco^TM^, Thermo Fisher Scientific, Inc., Waltham, MA, USA), 10,000 units/mL of penicillin, 10,000 μg/mL of streptomycin, and 25 μg/mL of Amphotericin B (Gibco^TM^, Thermo Fisher Scientific, Inc., Waltham, MA, USA), and then incubated at 37 °C with 5% CO_2_. The medium was changed twice weekly to remove nonadherent cells. Upon reaching 70–80% confluency, cells were trypsinized and passaged for expansion. Once sufficient cell numbers were reached, the cells were trypsinized, washed with DPBS, and counted using a hemocytometer. Cell viability was assessed via the trypan blue exclusion assay, as previously described [16]. Cells were resuspended in CryoStor^®^ CS10 (Biolife Solutions, Inc., Washington, DC, USA) and aliquoted into cryovials, which were pre-cooled at −80 °C overnight and then stored in liquid nitrogen. For use, MSCs were thawed at 37 °C, diluted, washed with DPBS, and assessed to ensure ≥95% viability prior to being resuspended in a sterile solution for injection within 4 h.

#### 2.3.2. cAD-MSC Characterization

The criteria for MSC identification were modified from the International Society for Cell & Gene Therapy (ISCT) [17].

(1) Flow cytometry

Cultured cells at passage 1 (1 × 10^6^ cells) were resuspended in PBS and stained with fluorochrome-conjugated antibodies to evaluate cell surface markers. For positive markers, cells were incubated with PE-labeled anti-Dog CD90 (eBioscience^TM^, Thermo Fisher Scientific, Waltham, MA, USA) and APC-labeled anti-Human CD105 (BioLegend^®^, San Diego, CA, USA). For negative markers, cells were stained with PE Mouse Anti-Dog CD34 (BD Pharmingen^TM^, BD Biosciences, San Jose, CA, USA) and PE-labeled anti-Human HLA-DR (BioLegend^®^, San Diego, CA, USA). All antibody incubations were performed separately in flow cytometry tubes for 30 min at 4 °C. Following incubation, cells were washed with PBS and analyzed for cell surface marker expression using a FACSCalibur flow cytometer (BD Biosciences, San Jose, CA, USA). Flow cytometric data were acquired and analyzed using CELLQuest Pro software (BD Biosciences, San Jose, CA, USA). All procedures were performed in accordance with the manufacturer’s instructions.

(2) In vitro differentiation assessment (Tri-differentiation)

The differentiation potential of the cells into osteoblasts, adipocytes, and chondrocytes was assessed using an Osteogenesis Differentiation Kit, an Adipogenesis Differentiation Kit, and a Chondrogenesis Differentiation Kit (StemPro^®^, Life Technologies, Carlsbad, CA, USA). Following the induction of differentiation, osteogenic lineage was confirmed by Alizarin Red staining, adipogenic lineage was confirmed by Oil Red O staining, and chondrogenic lineage was confirmed by Alcian Blue staining. Control cultures were included for each lineage. All procedures were performed in accordance with the manufacturer’s instructions.

(3) Sterility test

During cell culture, supernatants from the culture medium were collected and analyzed for microbial contamination. The samples were transferred to appropriate media vials for microbial detection: BD BACTEC^TM^ Plus Aerobic/F for aerobic bacterial growth, BD BACTEC^TM^ Lytic/10 Anaerobic/F for the detection of anaerobic bacteria, and BD BACTEC^TM^ Mycosis IC/F for fungal microorganisms (BD Biosciences, San Jose, CA, USA). These vials were subsequently processed using a BD BACTEC^TM^ Blood Culture System (BD Biosciences, San Jose, CA, USA) to detect the presence of bacteria and fungi. For mycoplasma contamination, polymerase chain reaction (PCR) was performed using an EZ-PCR^TM^ Mycoplasma Detection Kit (Biological Industries, Sartorius, Göttingen, Germany). Endotoxin levels were assessed using the kinetic chromogenic Limulus Amebocyte Lysate (LAL) assay (Endosafe^®^ nexgen-PTS, Charles River, Charleston, SC, USA). All tests were conducted in accordance with the manufacturer’s instructions.

### 2.4. Therapy with cAD-MSCs

Dogs were sedated with acepromazine maleate (Combistress^®^, KELA N.V., Hoogstraten, Belgium) (0.03 mg/kg), and 0.5% tetracaine hydrochloride (Alcon Laboratories Ltd., Bangkok, Thailand) was topically administered. The corneal wound was prepared by gently removing the loose corneal epithelium from the wound edge area using a sterile cotton ball. Dogs received a single subconjunctival injection of cAD-MSCs 1 × 10^6^ cells in 0.15 mL sterile solution at the central area of dorsal bulbar conjunctiva. Previous topical treatment associated with diamond burr debridement including 0.3% tobramycin eye drops (Tobrex^®^, Alcon, Bangkok, Thailand) and artificial tears containing hydroxypropyl methyl cellulose (Tear naturale^®^ II, Alcon Laboratories Ltd., Bangkok, Thailand) were continued four times daily throughout the experiment. The dogs were required to wear Elizabethan collars to avoid self-mutilation.

### 2.5. Evaluation of TNF-α and VEGF-A in Tear Fluid

#### 2.5.1. Tear Fluid Collection

Tear fluid was collected from all eyes before and after treatment on days 7, 14, and 21. First, 100 µL of tear samples was collected from the ventral conjunctival sac using a cellulose-based ophthalmic sponge (Weck-Cel ^®^ Sponge, Points and Strips, Beaver-Visitec International, Inc., Waltham, MA, USA) as previously described by Sebbag et al. [18]. The lower eyelid was manually pulled downwards. A 4 × 10 mm^2^ strip of cellulose-based ophthalmic sponge was inserted into the ventral conjunctival fornix using stainless tweezers and kept in place for 2 min. The wet sponge was transferred to a 0.2 mL Eppendorf tube and the tube was punctured using an 18-gauge needle to create drainage holes at the bottom of the tube. The 0.2 mL Eppendorf tube containing a wet sponge was transferred to a 1.5 mL Eppendorf tube and spun at 6000 revolutions per minute (rpm) for 1 min in a centrifuge machine. After centrifugation, the tear sample from the wet sponge was eluted through the drainage holes into a 1.5 mL Eppendorf tube. Finally, the 1.5 mL Eppendorf tube containing the tear fluid sample was stored at −80 °C for further analysis.

#### 2.5.2. Multiplex Cytokines Immunoassay

Tear fluid samples were assessed for TNF-α and VEGF-A concentrations using a commercially available canine cytokine magnetic bead panel immunoassay for TNF-α, and VEGF-A (Canine Procarta Plex^TM^ 11-plex immunoassay, Cat No EPX11A-50511-901, Thermo Fischer Scientific, Waltham, MA, USA). The test was performed according to the manufacturer’s instructions. Briefly, 10 μL of each tear sample was diluted in 40 μL of assay buffer (UAB, Thermo Fischer Scientific, Waltham, MA, USA) to obtain 50 μL of analyte (1:5 dilution as modified from the study by Gao et al. [19]). Then, 50 μL of Capture Bead Mix of TNF-α and VEGF-A was added to the 96-well plate, and the plate was washed twice using a handheld magnetic plate washer. Diluted samples, standards, and control solutions (50 μL each) were added in duplicates to the appropriate wells. The plate was placed on a plate shaker (600 rpm) for 2 h at room temperature and washed twice. Next, 25 μL of biotinylated detection antibodies (Thermo Fischer Scientific, Waltham, MA, USA) was added to each well. The plate was shaken at 600 rpm for 30 min at room temperature and washed twice. Next, 50 μL of Streptavidin/phycoerythrin (Thermo Fischer Scientific, Waltham, MA, USA) was added to each well, and the plate was placed on the shaker for 30 min and then washed twice. Then, 120 μL of the assay buffer was added to each well. The plates were shaken at 600 rpm for 5 min at room temperature. The plate was analyzed on a Luminex MAXPIG^®^ analyzer (Thermo Fischer Scientific, Waltham, MA, USA) using a five-parameter regression formula relative to the standard curve. The estimated minimum detectable concentration for each cytokine provided by the manufacturer was as follows: 5.1 pg/mL for TNF-α and 7.5 pg/mL for VEGF-A.

### 2.6. Data Analysis

#### 2.6.1. Evaluation Corneal Characteristics

Photographs of the cornea were taken at standardized magnification using a digital camera (Canon PowerShot G6; Tokyo, Japan) on day 0, 7, 14, and 21. Negative corneal fluorescein staining associated with non-epithelial lip indicated complete corneal re-epithelization. The areas of corneal epithelial defects [13], neovascularization [20], and opacification [5] were quantitatively analyzed using ImageJ software (ImageJ 1.31v software), and the results were expressed as a percentage of corneal lesions using equations as followed.

Corneal epithelial defect area (%) = [corneal epithelial defect area/total corneal area] × 100

Corneal neovascularization area (%) = [corneal neovascularization area/total corneal area] × 100

Corneal opacification area (%) = [corneal opacification area/total corneal area] × 100

The normality of the data distribution was assessed using the Shapiro–Wilk test. The percentages of corneal epithelial defects and corneal neovascularization areas were compared between different time points using the Friedman test together with the Bonferroni post hoc test, while the percentage of corneal opacification area was compared between different time points by repeated measures analysis of variance (ANOVA) with the Bonferroni post hoc test. All tests were performed using SPSS software (IBM SPSS Statistics 28.0.0.0). Statistical significance was defined as *p* < 0.05.

#### 2.6.2. Tear Fluid Cytokine Quantification

The normality of the data distribution was assessed using the Shapiro–Wilk test.

The concentrations of tear TNF-α and VEGF-A were compared between different time points using repeated ANOVA together with the Bonferroni post hoc test. All tests were performed using SPSS software (IBM SPSS Statistics 28.0.0.0). Statistical significance was defined as *p* < 0.05.

## 3. Results

Demographic data of canine eyes with SCCEDs are described in Table 1. Mean (± SEM) age of dogs was 10.2 ± 1.5 years with a range of 1 to 15 years of age.

### 3.1. Corneal Evaluation

After the subconjunctival injection of cAD-MSCs, no adverse effects were observed in any of the eyes throughout the experiment. The degree of ocular discomfort assessed by blepharospasm, photophobia, and lacrimation [21] gradually decreased over time. Ocular discomfort was absent in five eyes at day 7, eight eyes at day 14, and nine eyes at day 21. After treatment, 5/10 eyes achieved complete corneal healing within 7 days, while 3/10, 1/10, and 1/10 eyes completed corneal healing by day 14, 21, and 35, respectively (Supplementary Materials Table S1: Ophthalmic examinations of canine SCCEDs eyes before and after treatment).

#### 3.1.1. Corneal Epithelial Defect

**Figure 1:**
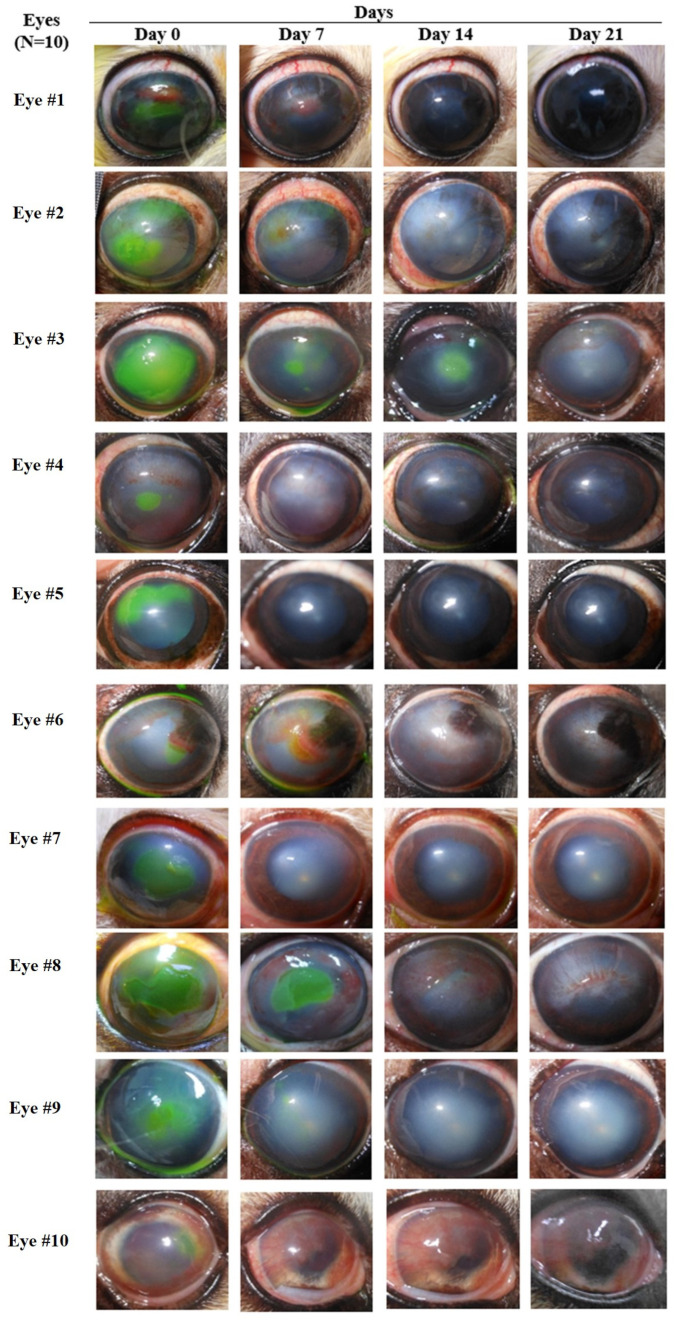
Corneal characteristics of 10 canine eyes with SCCEDs before and after treatment with the subconjunctival injection of cAD-MSCs.

**Figure 2:**
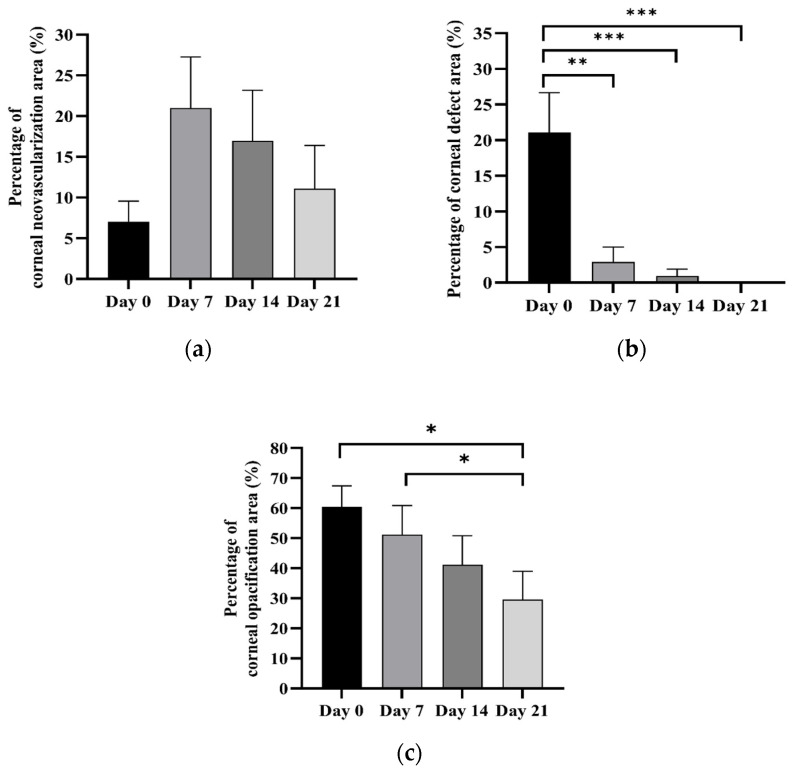
Bar graph illustrating percentage of corneal epithelial defect (**a**), corneal neovascularization (**b**), and corneal opacification area (**c**) before and after subconjunctival injection of cAD-MSCs; * *p* < 0.05, ** *p* < 0.01, *** *p* < 0.001.

Strengthening of the corneal epithelial wound edge was observed after treatment. The area of the corneal epithelial defect had obviously decreased by day 7 and then continued to decrease over time (Figure 1). The mean percentage of the corneal epithelial defect area before treatment was 21.09 ± 5.58. After cAD-MSCs treatment, at days 7, 14, and 21, the mean percentages of corneal epithelial defect statistically decreased to 2.91 ± 2.08, 0.95 ± 0.95, and 0, respectively, as shown in Figure 2a.

#### 3.1.2. Corneal Neovascularization

Before treatment, corneal vessels were observed in five of ten eyes. After treatment, corneal neovascularization occurred in another four eyes. New vessels generated from the corneal limbus, continuing to extend, branch, and finally reach the corneal wound edge. Three eyes showed circumferential vessels on the wounds, which markedly regressed thereafter (Figure 1). Only one eye presenting avascular cornea before treatment did not show corneal neovascularization, regardless of complete corneal re-epithelialization by day 7. The mean percentage of corneal neovascularization area before treatment was 7.02 ± 2.68. After cAD-MSCs treatment, it greatly increased to 18.90 ± 6.72 at day 7, then slightly decreased to 17.18 ± 6.05 at day 14 and then to 8.86 ± 4.89 at day 21, as shown in Figure 2b. No statistically significant differences were observed before and after treatment.

#### 3.1.3. Corneal Opacification

Corneal opacification was observed around the wounds in all eyes. The corneal opacity area decreased continuously after treatment (Figure 1). The mean percentage of the corneal opacification area before treatment was 58.44 ± 5.98. After cAD-MSCs treatment, it gradually decreased to 47.94 ± 8.28, 38.18 ± 8.16, and 25.78 ± 7.12 at days 7, 14, and 21, respectively (Figure 2c). Even though corneal opacification persisted at the end of the study, a statistically significant reduction was revealed between day 0 and day 21 and between day 7 and day 21.

### 3.2. Quantification of TNF-α and VEGF-A Concentrations in Tear Fluid

**Figure 3:**
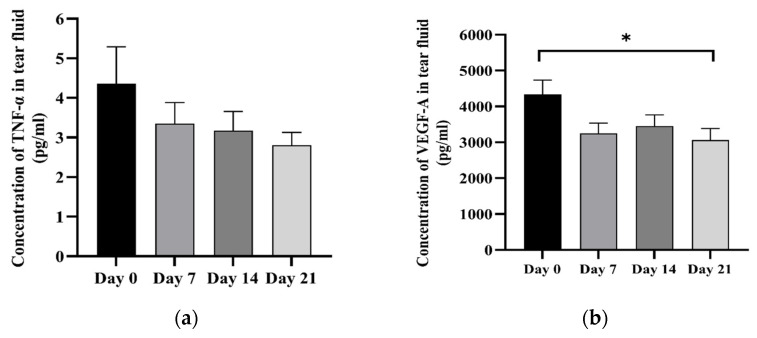
Bar graph illustrating concentrations of TNF-α (**a**) and VEGF-A (**b**) (pg/mL) in tear fluid before and after subconjunctival injection of cAD-MSCs; * *p* < 0.05.

TNF-α was detected in 97.5% of the tear fluid samples. The mean TNF-α concentration before treatment was 4.36 ± 0.93 pg/mL. After treatment, it was 3.35 ± 0.53, 3.17 ± 0.48, and 2.81 ± 0.32 pg/mL on days 7, 14, and 21, respectively (Figure 3a). There were no statistically significant differences in TNF-α concentration between before and after treatment. VEGF-A was detected in all tear fluid samples. The mean concentration of VEGF-A before treatment was 4334.91 ± 1275.92 pg/mL. After treatment, it declined to 3252.06 ± 898.30, 3450.78 ± 990.18, and 3064.61 ± 1028.66 pg/mL at days 7, 14, and 21, respectively (Figure 3b). A statistically significant difference was revealed between day 0 and day 21.

## 4. Discussion

To date, no published data have been evaluated detailing corneal outcomes and quantified tear cytokines in canine SCCEDs. SCCEDs are considered complicated corneal ulcers consisting of a hyalinized acellular zone, which is a barrier to reform corneal adhesion. Although diamond burr debridement significantly decreased the thickness of the hyalinized acellular zone in the anterior stroma [3], non-healed corneas were still evident. We herein demonstrated that in canine SCCEDs nonresponsive to two rounds of diamond burr debridement a significant decrease in corneal epithelial defect occurred following a single subconjunctival injection of cAD-MSCs. Ocular adverse effects from the treatment were not evident, as reported in other animal model studies [13,14,15,22]. Though there are currently no reports of concerned abnormalities from MSCs, conducting follow-up assessment is imperative for ethical considerations.

The mean corneal healing time at the day of examination in this current study (10.89 ± 1.7 days) was slightly shorter than that in a report from Falcao et al. (12.73 ± 0.59 days) performed on various types of corneal ulcers [15]. In their study, all dogs received a combined treatment of subconjunctival injection and topical instillation of cAD-MSCs for a total of 3 × 10^6^ cells. Topical application is the most common route for the application of MSCs because of the ease of application. However, Shukla et al. [5] investigated the therapeutic effects of different routes of the administration of bone marrow-derived mesenchymal stem cells (BM-MSCs), including topical, subconjunctival, intravenous, and intraperitoneal, following corneal mechanical injury in mice. The results demonstrated that subconjunctival and intravenous routes of MSC administration were greatly effective in promoting corneal re-epithelization based on the percentage of corneal defect area. Moreover, histopathological results revealed a reduction in inflammatory cells in the cornea following subconjunctival delivery compared with other routes. In this study, we demonstrated that the subconjunctival injection of 1 × 10^6^ cAD-MSCs is adequate to achieve complete corneal healing in cases of canine SCCEDs that were previously unresponsive to conventional treatment options. However, there is a possibility that corneal wound healing may have been completed before days of examination. More frequent post-treatment examinations may be necessary to investigate an exact day of corneal wound healing.

Delayed corneal re-epithelialization occurred in one eye with a prolonged presence of a loosened epithelial wound edge. Complete corneal wound healing occurred by day 35 after treatment without additional intervention. This dog concurrently presented with atopic dermatitis, which may have influenced delayed corneal wound healing. Canine atopic dermatitis is a common chronic inflammatory skin disease occasionally associated with ocular surface inflammation. A preliminary study of cytokines on the ocular surface of atopic dogs reported that IL-8, a pro-inflammatory cytokine, is overexpressed in the conjunctiva and tears [23]. The study by Fukada et al. [24] investigated the effect of allergic inflammation in the conjunctiva on corneal epithelial wound healing in a rat model. Corneal epithelial wound healing is significantly delayed by conjunctival allergic inflammation associated with increased eosinophil infiltration into the conjunctiva in vivo. Furthermore, conjunctival allergic inflammation is associated with ocular allergic diseases such as vernal keratoconjunctivitis and corneal diseases such as persistent epithelial defects and shield ulcers in humans [25].

There is evidence suggesting that MSCs administered subconjunctivally can migrate to the injured cornea. The presence of MSCs, labeled with the fluorescent dyes CM-DiI [12] and Qdots [5], was observed on the cornea using confocal microscopy. Furthermore, there was a study reporting that MSCs promote corneal epithelial healing by reducing the levels of pro-inflammatory cytokines, particularly TNF-α. Ke et al. [3] demonstrated that TNF-α expression in the corneas of chemically induced rats was high, after which it gradually decreased after the subconjunctival injection of BM-MSCs during the corneal healing process, as compared to the controls. Similarly, after the subconjunctival injection of BM-MSCs in mice with mechanically injured corneas, the expression of TNF-α in the corneas was suppressed [12]. The median concentration of TNF-α in healthy canine tears was 3.5 pg/mL [26]. Dogs presenting with ocular combined with dermatologic disorders had an increased median value to 6.1 pg/mL. Our study delineated a correlation in TNF-α concentration levels, corroborating the findings reported by Martinez et al. [26]. The highest mean concentration of TNF-α was observed on day 0, followed by a progressive decrease that culminated in the lowest concentration on day 21, which corresponded with the complete re-epithelialization of the cornea. Although our study demonstrated continuous reduction in the TNF-α level in the tear fluid of canines with SCCEDs, there was no statistically significant difference after cAD-MSCs injection. It is advised that other inflammatory cytokines should be assessed to gain further insights. Furthermore, all eyes received dry debridement to remove loosened corneal epithelium and a continuous topical medication. It is still controversial to conclude that complete epithelialization is solely the result of cAD-MSCs. To gain better understanding of corneal cell adhesion, transmission electromicroscopy of canine corneas with SCCEDs after cAD-MSCs treatment is suggested for future investigation.

MSCs are widely known to promote angiogenesis in skin wound healing [27], brain injury [28], and myocardial infarction [29]. In contrast with other organs, MSCs have an anti-angiogenic effect in the cornea via direct cell-to-cell interaction or by paracrine activity [2,13,30,31]. According to a previous study, corneal vascularization was observed in 58–64% of canine SCCEDs [9]. This finding was consistent with our study in that corneal vascularization was observed in only 50% of the eyes before treatment. Based on our results, the area of corneal vascularization was greatest on day 7 after treatment and then gradually decreased until the end of the study, while the concentration of tear film VEGF-A, a cytokine involved in the promotion of corneal angiogenesis, was constantly decreased after treatment. Although no statistically significant difference was observed in the corneal neovascularization area between all the time points in our study, we reported results that were consistent with the study of Ke et al. [3]. They investigated the efficacy of the subconjunctival injection of BM-MSCs on corneal alkali burns in rats. Their MSC group did not show corneal neovascularization on day 0 after injury. Interestingly, corneal vascularization continually increased from days 7 and then reached a peak on day 14 before beginning to decline. They also investigated VEGF concentration. They found the upregulation of VEGF on day 3 after corneal injury, which then gradually decreased. It is therefore speculated that the initial increase in corneal vascularization may be attributed to the corneal response from alkaline injury, which is a similar condition to the initial presence of corneal vessels in our study from mechanical removal of the loosened corneal epithelium. Assessing VEGF concentrations before day 7 post-cAD-MSC treatment could offer more understanding of corneal neovascularization that may be related to an alteration VEGF concentration. Furthermore, in our study, the mean concentration of VEGF-A following complete corneal healing was determined to be 3064.61 ± 1028.66 pg/mL. In contrast, the mean VEGF-A concentration in healthy canine tears was approximately 200 pg/mL [32]. Following the induction of conjunctivitis via histamine administration, a dose-dependent increase in VEGF-A concentration was observed. Consequently, several factors, including the dilution of tear samples, methodologies for tear collection, storage conditions, and the underlying pathophysiological mechanisms, may contribute to the observed variability in VEGF-A concentrations.

In this study, we demonstrated that the corneal opacification area gradually decreased after injection with cAD-MSCs, which is consistent with the study by Shukla et al. [5]. They demonstrated that after the subconjunctival injection of BM-MSCs on corneal injuries in mice, the percentage of corneal opacification area decreased, together with a significantly reduced expression of α-Sma and TGF-β compared to the untreated group. Following corneal injury, the upregulation of TGF-β promotes the conversion of quiescent stromal keratocytes into α-Sma-containing myofibroblasts. Excessive accumulation of myofibroblasts and their activity results in the development of corneal opacity and scarring. In addition, corneal opacity is mainly affected by corneal edema in the early phase of corneal injury, followed by fibrosis in the later phase [33]. Corneal edema occurs by osmotic absorption of fluid from the tear film during corneal epithelial damage. Thus, excessive fluid should be removed by the endothelium after corneal re-epithelialization. Consequently, this mechanism may contribute to the reduction in the mean corneal opacification area after treatment with cAD-MSCs in our study. However, corneal opacity partially remained in some eyes by the end of the study. This remaining corneal opacity may be related to corneal fibrosis from the chronic stage of canine SCCEDs, which requires time for corneal remodeling.

## 5. Conclusions

In summary, a single subconjunctival injection of 1 × 10^6^ cAD-MSCs could be used as an alternative treatment for canine SCCEDs that were nonresponsive to diamond burr debridement. It is a noninvasive therapy with no adverse effects observed during this study.

## Figures and Tables

**Table 1:** Demographic data of canine eyes with SCCEDs.

Eye Number	Eye	Breed	Age (Years)	Sex	Concurrent Ocular Diseases	Other Diseases	Presence of Corneal Healing (Days)
1	OD	Chihuahua	1	F	-	-	7
2	OD	Shih tzu	12	F	Mild KCS	-	14
3	OD	Yorkshire terrier	15	F	Mild KCS	AD	21
4	OD	Chihuahua	5	M	-	-	7
5	OS	Boston terrier	10	F	-	-	7
6	OD	Shih tzu	13	F	Moderate KCS	-	14
7	OS	Shih tzu	15	F	Moderate KCS	-	7
8	OD	French bulldog	8	M	-	AD	14
9	OS	Yorkshire terrier	15	F	Mild KCS	AD	7
10	OD	Siberian husky	3	F	-	OE, AD	35

Note: Presence of corneal healing means the observation of complete corneal wound healing at days of examination after treatment; OD = Oculus Dexter; OS = Oculus Sinister; KCS = Keratoconjunctivitis Sicca; AD = atopic dermatitis; OE = Otitis externa; F = female; M = male.

## Data Availability

Data are contained within the article and Supplementary Materials.

## Supplementary Materials

The following supporting information can be downloaded at: https://www.mdpi.com/article/10.3390/ani14223270/s1, Table S1: Ophthalmic examinations of canine SCCEDs eyes before and after treatment.
