# Supplementary material for: Effect of Subconjunctival Injection of Canine Adipose-Derived Mesenchymal Stem Cells on Canine Spontaneous Corneal Epithelial Defects
Source: Animals (Basel). 2024 Nov 13;14(22):3270. doi: 10.3390/ani14223270 (PMC11591453; doi:10.3390/ani14223270)
Supplement: Supplementary file 1 [file animals-14-03270-s001.zip › animals-3241996-supplementary.pdf]

Table supplement: Ophthalmic examinations of canine SCCEDs eyes before and after treatment.

Eye number	Examination Day	STT (mm/min)	Fluoresceine staining	Presence of nonadherent epithelium	IOP (mmHg)	Duration of SCCEDs (week)	Location of SCCEDS
1	0	22	+	+	15	3	Center
	7	18	-	-	13		
	14	16	-	-	14		
	21	15	-	-	13		
2	0	14	+	+	16	4	Center, Quadrant 1, 3, 4
	7	14	+	-	14		
	14	12	-	-	14		
	21	12	-	-	15		
3	0	18	+	+	11	6	Center Quadrant 1, 2, 3, 4
	7	14	+	-	10		
	14	13	+	-	12		
	21	13	-	-	14		
4	0	21	+	+	17	3	Center
	7	16	-	-	15		
	14	15	-	-	15		
	21	15	-	-	16		
5	0	25	+	+	11	3	Quadrant 1, 2
	7	17	-	-	10		
	14	15	-	-	13		
	21	16	-	-	15		
6	0	20	+	+	14	6	Center
	7	16	+	+	15		
	14	11	-	-	18		
	21	13	-	-	16		
7	0	14	+	+	12	4	Center
	7	13	-	-	12		
	14	12	-	-	14		
	21	12	-	-	12		
8	0	23	+	+	15	6	Center
	7	20	+	+	14		
	14	15	-	-	12		
	21	16	-	-	15		
9	0	16	+	+	15	3	Center
	7	14	-	-	14		
	14	14	-	-	16		
	21	13	-	-	16		
10	0	18	+	+	13	8	Center Quadrant 1
	7	15	-	+	11		
	14	14	-	+	14		
	21	12	-	+	14		

Note: Duration of SCCEDs refers to the duration of the disease before MSC being administered. # Location of SCCEDs is divided into 5 areas: center, quadrant 1 (12 to 3 o'clock), quadrant 2 (9-12 o'clock), quadrant 3 (6-9 o'clock), and quadrant 4 (3-6 o'clock).
